# Hospitalizations for viral respiratory infections in children under 2 years of age: epidemiology and in-hospital complications

**DOI:** 10.1186/s12887-020-02186-7

**Published:** 2020-06-09

**Authors:** Jessie N. Zurita-Cruz, Alejandro Gutierrez-Gonzalez, Leticia Manuel-Apolinar, José Esteban Fernández-Gárate, María Luisa Arellano-Flores, Roberto Alejandro Correa Gonzalez, Guillermo Vázquez-Rosales, Rocio Sanchez-Armas, Nelly Cisneros-González

**Affiliations:** 1 School of Medicine, Universidad Nacional Autónoma de Mexico, Mexico City, Mexico; 2 Hospital Infantil de Mexico Federico Gómez, Mexico City, Mexico; 3 Unit of Research in Medical Nutrition, Pediatric Hospital “Centro Médico Nacional Siglo XXI”, Instituto Mexicano del Seguro Social (IMSS), Mexico City, Mexico; 4 Computer Research Center of Instituto Politecnico Nacional, Mexico City, Mexico; 5 Endocrine Research Unit, Centro Médico Nacional, Instituto Mexicano del Seguro Social (IMSS), Mexico City, Mexico; 6 Epidemiological Surveillance Coordination, Instituto Mexicano del Seguro Social (IMSS), Mexico City, Mexico; 7 Health Research Coordination, Instituto Mexicano del Seguro Social (IMSS), Mexico City, Mexico; 8 Infectology Department, Pediatric Hospital “Centro Médico Nacional Siglo XXI”, Instituto Mexicano del Seguro Social (IMSS), Mexico City, Mexico

**Keywords:** Respiratory tract infection, Viruses, Epidemiology, Bronchiolitis, Mexico

## Abstract

**Background:**

Viral respiratory infections (VRIs) are a frequent cause of hospitalization in children under 24 months of age. A history of prematurity or heart disease may be a risk factor for complications in patients hospitalized for VRI. The objective was to describe epidemiological data for children hospitalized for IRV and aged 1 to 24 months and to identify risk factors for the presence of in-hospital complications and mortality over a period of 5 years.

**Methods:**

This was a cross-sectional study. Patients registered with VRI codes B974, J12, J120-J129X, J168, J17, J171, J178, J20, J203-J209, J21, J210, J211, J218, J219 (based on International Classification of Diseases [ICD-10]) from 2013 to 2017 were included. Three subanalyses were performed to compare [1] patients with pathological history (prematurity, bronchopulmonary dysplasia [BPD] and congenital heart disease [CHD]), [2] diagnoses (pneumonia, acute bronchitis, and acute bronchiolitis), and [3] admission to the pediatric intensive care unit. Days of hospital stay, in-hospital complications, invasive medical procedure and mortality were analyzed. Statistical analysis**:** VRI hospitalization prevalence was described. For comparison between groups, Student’s t-test, ANOVA and the Chi2 test were applied. To identify factors related to days of hospital stay, in-hospital complications and mortality, a linear and logistic regression model was performed.

**Results:**

A total of 66,304 hospitalizations were reported. The average age was 14.7 weeks; hospitalization events were higher in winter (39%), followed by autumn (27.3%). A total of 371 (0.56%) patients died. A total of 7068 (10.6%) hospitalized patients with pathological histories were identified. The presence of BPD (coefficient = 1.6), CHD (coefficient = 1.2), diagnosis of pneumonia (coefficient = 1.2), in-hospital complications (coefficient = 2.1) and invasive medical procedures (coefficient = 15.7) were the most common factors that increased the length of hospital stay. Risk factors for in-hospital complications and mortality were invasive medical procedure (OR = 3.3 & 11.7), BPD (OR = 1.8 & 1.6), CHD (OR = 4.6 & 3.4) and diagnosis of pneumonia (OR = 1.8 & 4.2).

**Conclusions:**

Risk factors for morbidity and mortality in patients hospitalized for VRIs are BPD and CHD, diagnosis of pneumonia and invasive medical procedure.

## Background

Viral respiratory infections (VRIs), including bronchiolitis, are the main cause of hospitalization in pediatric patients under 2 years of age [1]. It is estimated that hospital expenses secondary to this disease reach up to $1.4 billion, constituting a significant public health burden [2]. Research on hospitalizations for VRI have shown that the main etiologic agent is respiratory syncytial virus (RSV) [3].

A complete clinical examination is indispensable in the initial evaluation of patients. Evidence of inadequate feeding or fluid intake, history of apnea, lethargy, moderate to severe respiratory distress (nasal flutter, tachypnea, respiratory whining, retractions or cyanosis) and low oxygen saturation at ambient air require hospitalization [3, 4]. Therefore, admission to the pediatric intensive care unit (PICU) in patients with clinical signs of exhaustion, markers of acute respiratory failure or apnea should be considered [5]. In general, an etiological diagnosis is made with clinical data, and an age less than 2 years invokes high suspicion of RSV [6].

Patients with a history of prematurity have a higher risk of complications in VRIs due to a lack of lung development and an inadequate immune system response. The condition of bronchopulmonary dysplasia (BPD), in which structural abnormalities persist in the lungs, is also a complication that results in greater complexity and vulnerability of torpid evolution presentation in VRI [7–10]. Another group of patients with a high risk of mortality secondary to VRI are those diagnosed with congenital heart disease (CHD), which may be related to multiple physiological factors, including baseline compromised cardiorespiratory function, changed mechanisms of pulmonary regulation, ventilation – perfusion mismatch, pulmonary hypertension and/or an inadequate immune response secondary to malnutrition that some patients with CHD may present [11, 12].

To date, there are no updated epidemiological data for this disease in Mexico or information about the risk factors related to hospital complications in our population. The objective of our study was to describe the epidemiological data for children hospitalized for VRI and aged from 1 to 24 months and to identify risk factors for the presence of in-hospital complications and mortality in beneficiaries of the Mexican Social Security Institute (IMSS) over a period of 5 years (January 2013 to December 2017).

## Methods

This was a cross-sectional study. The patients were clinically diagnosed by doctors at IMSS. The data used in this study were obtained from the System of Information of Integral Health Care and the hospital records of the System of Medical Statistics (DataMart) of the IMSS and the databases of Family Medicine Units. At the IMSS, medical attention is classified into three levels: 1st-level facilities perform preventive measures and treat acute and chronic pathologies without complications; 2nd-level facilities address complicated pathologies, chirurgic interventions, and treatments that require hospitalization; and 3rd-level facilities are equipped to treat individuals with complex and complicated diseases. At IMSS database, each patient is identified uniquely, and his/her record is updated accordingly across all clinics the patient may have been treated. Diseases are registered through the codes of the International Classification of Diseases (ICD 10).

The data in the database were compiled by doctors who attend and diagnose patients visiting hospitals affiliated with the IMSS; the data are validated by a team of engineers in computer systems and are analyzed by statisticians affiliated with specialized centers strategically located at regional and state levels.

For this study, patients aged 1 to 24 months hospitalized for severe acute respiratory infection with respiratory distress, febrile syndrome, inadequate oral intake or dehydration from January 2013 to December 2017 in IMSS hospitals were included. Patients included were described using the following ICD-10 codes: *B974,* which includes RSV; *J12*, which includes viral *(J120-J129X)* or nonspecific *(J168, J17, J171, J178)* pneumonia, acute bronchitis *(J20, J203-J209),* and acute bronchiolitis *(J21, J210, J211, J218, J219).*

The study included patients who, according to records, were first hospitalized at the IMSS for respiratory tract infections.

Patients with immunodeficiency *(D81-D84)*, neurological disorders *(G40-G41, G71, G80-G83)*, airway malformations *(Q30-Q34)* or incomplete data were excluded.

In-hospital complications were defined as any event or condition detrimental to the patient’s health, as caused by an unintentional injury and recorded by the medical and nursing staff during hospitalization. In-hospital complications were grouped and labeled as infectious, respiratory, metabolic and cardiovascular complications.

In addition, codes for respiratory complications such as nonviral pneumonia *(J13, J13X, J14, J15, J151, J152, J156, J158, J159, J16, J181, J182)*, respiratory failure *(J96, J960, J969)*, and nonspecific respiratory disorder *(J988, J989)* were included in the diagnoses.

An invasive medical procedure was defined as deliberate access to the body through an incision or a percutaneous puncture, where instrumentation is used in addition to the puncture needle or instrumentation through a natural orifice, and performed by trained professionals who use instruments; only invasive procedures related to VRI were included.

Patients were classified according to pathological history, including history of BPD, prematurity and CHD.

A history of prematurity was identified by relevant ICD-10 codes *(P070, P072, P073).* The patients were subclassified into premature and extremely premature groups according to the history of weeks of gestational age (wGA). Prematurity was defined as 29–36 wGA at birth and extremely premature as < 29 wGA at birth. The presence of BPD was identified by code *P271,* and congenital heart disease with hemodynamic compromise was identified by codes *Q20-Q26*.

The criteria for severe acute respiratory infection were considered when the patient required endotracheal intubation and was admitted to the PICU. A subanalysis of the patients admitted to the PICU was performed.

Of the included patients, data regarding sex, age, location, month of hospitalization, days of hospital stay and complications during stay were collected, as was the reason for hospital discharge.

The primary outcomes that were included were the length of hospital stay, in-hospital complications, invasive medical procedure and mortality.

### Statistical analysis

Quantitative variables are presented as the mean, standard deviation, minimum and maximum. Qualitative variables are presented as proportions and frequencies. Three subanalyses were performed to compare (1) patients with pathological history (prematurity, BPD and CHD), (2) admission diagnoses (pneumonia, acute bronchitis, and acute bronchiolitis), and (3) admission to the PICU.

A nonnormal distribution was observed for quantitative variables (age and length of hospital stay) using the Kolmogorov-Smirnov test; therefore, log-transformation was used for the statistical analysis. Student’s t-test, ANOVA, and χ2 analyses were applied for inferences.

To identify factors related to the length of hospital stay, multiple linear regression was performed; the linear regression model met the assumptions of linearity, normality, homoscedasticity and independence. To identify factors related to in-hospital complications and mortality, a multiple logistic regression model was constructed with “step backward” modeling, and a fitting model was obtained, whereby the noncollinearity of the variables was confirmed.

A value of *p* < 0.05 was considered statistically significant.

STATA v.12.0 was used for the statistical analyses.

The protocol was approved by the National Research Ethics Committee, which belongs to the Mexican Institute of Social Security. This committee is the body in charge of evaluating research projects at the national level.

## Results

From 2013 to 2017, 66,304 patients from 1 month to 24 months of age were hospitalized due to VRI. For the general characteristics of the population, a male predominance was observed (61.1%), with an average age of 14.7 weeks (Table 1).

**Table 1:** General characteristics of 66,304 patients aged 1 to 24 months hospitalized for viral respiratory infections from 2013 to 2017 in the IMSS

		Frequency (%)
Age (weeks)^a^		14.7 ± 15.3 (5–102)
Sex Male		40,450 (61.01)
Hospitalization according to the season of the year	Spring	14,561 (21.96)
	Summer	7737 (11.67)
	Fall	18,093 (27.29)
	Winter	25,913 (39.08)
Hospitalization year	2013	17,378 (26.21)
	2014	13,185 (19.89)
	2015	13,773 (20.77)
	2016	10,810 (16.30)
	2017	11,158 (16.83)
Days of hospital stay ^a^		4.1 ± 4.4 (1–205)
Presence of pathological history		7068 (10.66)

^a^Mean ± standard deviation (minimum-maximum)

The average number of hospitalized patients per year was 13,260, with a gradual decrease in hospitalizations over the course of 5 years. Hospitalizations according to the season of the year were greater in winter (39.08%), followed by autumn (27.29%). The average hospital stay was 4.1 days, with a maximum stay of 205 days (Table 1). The most frequent hospitalization diagnosis was acute bronchiolitis (56.93% *n* = 37,747), followed by pneumonia (38.32%, *n* = 25,411) and acute bronchitis (4.74% *n* = 3146). A total of 7068 (10.66%) hospitalized patients with a pathological history were identified; 329 patients (0.50%) required a stay in the PICU due to the severity of the disease. Of the total hospitalized patients with a history of prematurity, 123 had a history of extreme prematurity, of which six (4.8%) required invasive medical procedures, two (1.6%) had in-hospital complications, and three (2.4%) required hospital stay in the PICU; three (2.4%) died.

During the hospital stay, a low frequency of complications was reported (1.22% *n* = 812), with respiratory complications being the most frequent (41.40% *n* = 336). Invasive medical procedures were performed for 552 patients (0.83%), and of these interventions, the most common was the placement of a venous catheter (*n* = 302), followed by endotracheal intubation (*n* = 170) and bronchoscopy with or without biopsy (*n* = 80) (Table 2).

**Table 2:** Hospital evolution of 66,304 patients aged 1 to 24 months due to viral respiratory infections from 2013 to 2017 in the IMSS

		Frequency (%)
In-hospital complications		812 (1.22)
Type of in-hospital complications	Respiratory	336 (41.40)
	Cardiovascular	240 (29.56)
	Metabolic	151 (18.60)
	Infectious	85 (10.44)
Pathological history	Prematurity	3234 (45.76)
	Bronchopulmonary dysplasia (BPD)	2467 (34.90)
	Congenital heart disease	762 (10.78)
	Congenital heart disease & prematurity	327 (4.63)
	Congenital heart disease & BPD	278 (3.93)
Invasive medical procedure		552 (0.83)
Admitted to Pediatric Intensive Care Unit		329 (0.50)
Mortality		371 (0.56)

*Mean ± standard deviation (minimum-maximum)

Mortality among the hospitalized patients was 0.56% (*n* = 371), with an average age of 13 weeks. The most frequent diagnosis was respiratory failure secondary to pneumonia (*n* = 182), followed by heart failure (*n* = 98), septic shock (*n* = 55), hydroelectrolytic imbalance (*n* = 20), and others (*n* = 6) (Table 2). Of the deceased patients, 38.8% (*n* = 144) had a pathological history (*n* = 89 with prematurity, *n* = 21 with BPD, *n* = 34 with CHD). Of the patients who died from respiratory failure, 19 had BPD. Among the patients with heart failure, 34 had CHD. In addition, of the 45 patients with septic shock, 35 had a history of prematurity, 3 had a history of extreme prematurity, and two had BPD.

Within this group of hospitalized patients, multiple factors were identified that might impact in-hospital complications and mortality. Therefore, a subanalysis according to the type of diagnosis at admission, presence of pathological history and stay in the PICU was performed. Regarding the admission diagnosis, the patients with a diagnosis of pneumonia had a longer hospital stay (pneumonia 5.09 vs. bronchitis 3 vs. bronchiolitis 3.5 days), a higher proportion of in-hospital complications (pneumonia 1.83% vs. bronchitis 0.45% vs. bronchiolitis 0.88%) and invasive medical procedures (pneumonia 1.64% vs. bronchitis 0.64% vs. bronchiolitis 0.31%), a longer stay in the PICU (pneumonia 1.07% vs. bronchitis 0.45% vs. bronchiolitis 0.11%) and higher mortality (pneumonia 1.14% vs. bronchitis 0.06% vs. bronchiolitis 0.21%) than patients who had bronchitis and bronchiolitis (Table 3). When dividing the patients according to the presence of a pathological history, the length of hospital stay (6.09 vs. 3.94 days), the presence of complications (5.8% vs. 1.31%), invasive medical procedure (0.67% vs. 2.52%) and death (0.4% vs. 2.16%) were greater in patients with pathological history than in those without them (Table 3). The patients who were admitted to the PICU had a longer hospital stay (21.6 vs. 4 days) and a higher proportion of in-hospital complications (6.69% vs. 1.20) and invasive medical procedures (100% vs. 0.34%) and mortality (9.12% vs. 0.52%) than patients who were not admitted to the PICU. None of these comparisons identified that age had an impact on the progression of the cases (Table 3).

**Table 3:** Comparison of the characteristics of patients with and without Pathological history, diagnosis at admission and admitted to Pediatric Intensive Care Unit from 1 to 24 months of age hospitalized for viral respiratory infections from 2013 to 2017 in the IMSS

	Pathological history			Diagnosis at admission				Admitted to Pediatric Intensive Care Unit		
	Without *n* = 59,236	With *n* = 7068	p	Pneumonia *n* = 25,411	Bronchitis *n* = 3146	Bronchiolitis *n* = 37,747	p	No *n* = 65,975	Yes *n* = 329	p
Frequency (%)										
Age (weeks)*	14.7 ± 15.3 (5–102)	14.6 ± 14.8 (5–102)	0.758	14.8 ± 16.0 (5–102)	14.3 ± 15.5 (5–102)	14.7 ± 14.7 (5–102)	0.786	14.7 ± 15.3 (5–102)	14.5 ± 14.5 (5–102)	0.832
Sex Male	36,283 (61.25)	4166 (58.94)	< 0.001	15,092 (59.39)	1875 (59.60)	23,482 (62.21)	< 0.001	36,283 (61.25)	4166 (58.94)	< 0.001
Hospital stay (days)*	3.9 ± 3.8 (1–139)	6.0 ± 7.6 (1–205)	< 0.001	5.09 ± 5.7 (1–205)	3.0 ± 3.4 (1–103)	3.5 ± 3.2 (1–119)	< 0.001	4.0 ± 4.0 1–205)	21.6 ± 22.1 (1–139)	< 0.001
In-hospital complications	540 (0.91)	272 (3.85)	< 0.001	466 (1.83)	14 (0.45)	332 (0.88)	< 0.001	790 (1.20)	22 (6.69)	< 0.001
Invasive medical procedure	373 (0.63)	179 (2.53)	< 0.001	416 (1.64)	20 (0.64)	116 (0.31)	< 0.001	223 (0.34)	329 (100)	< 0.001
Pediatric Intensive Care Unit	221 (0.37)	108 (1.53)	< 0.001	272 (1.07)	14 (0.45)	43 (0.11)	< 0.001	–	–	–
Mortality	227 (0.38)	144 (2.04)	< 0.001	290 (1.14)	2 (0.06)	79 (0.21)	< 0.001	341 (0.52)	30 (9.12)	< 0.001

Mean ± standard deviation (minimum-maximum)

To identify factors related to the length of hospital stay, several multivariate models were built. We observed that the presence of BPD (coefficient = 1.64, CI95% 1.37 to 1.90), CHD (coefficient = 1.24, CI95% 1.59 to 1.87), diagnosis of pneumonia (coefficient = 1.21, CI95% 1.14 to 1.27), in-hospital complications (coefficient = 2.19, CI95% 1.90 to 2.48) and invasive medical procedures (coefficient = 15.78, CI95% 15.24 to 16.32) were the most common factors that increased the length of hospital stay (Table 4).

**Table 4:** Multiple linear regression analysis to identify the factors related to the days of hospital stay of children 1 to 24 months of age hospitalized for viral respiratory infections (*n* = 66,304)

	Coefficient	Confidence interval 95%	p
Sex female	0.06	0.002 to 0.13	0.043
History of prematurity	0.55	0.408 to 0.707	< 0.001
Bronchopulmonary dysplasia	1.64	1.37 to 1.90	< 0.001
Congenital heart disease	1.73	1.59 to 1.87	< 0.001
In-hospital complications	2.19	1.90 to 2.48	< 0.001
Invasive medical procedures	15.78	15.24 to 16.32	< 0.001
Admitted to Pediatric Intensive Care Unit	0.78	0.08 to 1.48	0.029
Diagnosis at pneumonia	1.21	1.14 to 1.27	< 0.001

To identify factors that impact hospital complications, a multivariate model was evaluated, and it was observed that the most influential factors were invasive medical procedures (OR = 3.35 CI95% 2.35 to 4.77) and CHD (OR 4.69 CI95% 3.97 to 5.55), followed by BPD, a history of prematurity and diagnosis of pneumonia (Table 5.A).

**Table 5:** Logistic regression analysis to identify the factors that impact on in-hospital complications (A) and mortality (B) of children hospitalized for viral respiratory infections from 2013 to 2017 in the IMSS (*n* = 66,304)

A. Intrahospital complications			
	OR	Confidence interval 95%	p
History of prematurity	1.47	1.14 to 1.89	0.002
Bronchopulmonary dysplasia	1.83	1.32 to 2.53	< 0.001
Congenital heart disease	4.69	3.97 to 5.55	< 0.001
Invasive medical procedures	3.35	2.35 to 4.77	< 0.001
Diagnosis at pneumonia	1.84	1.59 to 2.12	< 0.001
B. Mortality			
	OR	Confidence interval 95%	p
Sex female	1.34	1.08 to 1.65	0.006
History of prematurity	1.24	0.84 to 1.82	0.409
Bronchopulmonary dysplasia	1.69	1.07 to 2.67	< 0.001
Congenital heart disease	3.40	2.65 to 4.37	< 0.001
In-hospital complications	7.87	5.75 to 10.78	< 0.001
Invasive medical procedures	11.17	8.14 to 15.33	< 0.001
Diagnosis at pneumonia	4.25	3.30 to 5.47	< 0.001

The multivariate model for factors that impact mortality showed that the most influential factors were invasive medical procedures (OR = 11.17, CI95% 8.14 to 15.33), diagnosis of pneumonia (OR 4.25, CI95% 3.30 to 5.47) and CHD (OR 3.40, CI95% 2.65 to 4.37), female sex and BPD (Table 5.B).

## Discussion

The analysis in our study allowed us to recognize that a history of CHD, BPD, diagnosis at pneumonia and invasive medical procedures were risk factors for the presence of in-hospital complications and mortality in children under 24 months of age hospitalized for VRI.

BPD is the most frequent chronic lung disease in infancy [13]; compared to healthy preterm infants, these patients have been shown to have fewer mature macrophages in the airways in the immediate perinatal period. The circulating monocytes of these patients also show decreased HLA-DR expression [14]. These conditions, both prematurity and BPD, render these patients more vulnerable to serious complications during VRI [7–10]. In our study group, BPD proved to be a risk factor for mortality (OR = 1.69), mainly because BDP has a mortality rate of 40 to 5%, depending on the wGA [15]. In patients with CHD and BPD who have an increased risk of presenting pulmonary hypertension due to their pathology, the presence of a VRI can worsen this secondary pulmonary hypertension due to multiple factors, including lung volume changes either with atelectasis or hyperinflation, hypoxic vasoconstriction, endothelin pathway, and Th2-skewed immune response [16–18]. This adverse environment that occurs in patients with CHD constitutes a risk for developing heart failure, which causes a longer hospital length of stay and increased mortality [16]. As we observed in this study, in which patients died of heart failure, 34.6% had a pathological history of CHD.

There are multiple factors associated with in-hospital complications in this group of patients, and prematurity has been demonstrated; the OR described in a meta-analysis was 1.96 (95% CI 1.44–2.67), which was greater than that in our study (OR = 1.47). Premature birth interrupts the transfer of maternal antibodies, and maturation of the immune system occurs at 6 months of life; thus, patients with a history of prematurity are susceptible to airway infections, particularly of viral etiology [19]. Furthermore, patents with a history of prematurity have acquired dysfunction of the autonomic nervous system that might result from an inflammatory cascade induced by a virus, which can cause apnea, increasing the risk of death [13, 20]. Additionally, patients with a history of premature birth present structural changes in the lung, including increased bronchial muscle, collagen, and elastin. Moreover, premature exposure to high oxygen tension and growth restriction as well as other aspects of the extrauterine environment worsen these effects [21, 22]. These conditions have shown, as in the results of our study, that patients with a history of prematurity have greater comorbidities in addition to a VRI greater severity and, therefore, a longer hospital stay [23].

The presence of some invasive medical procedure as a risk factor for poor prognosis can serve as an indirect indicator of the severity of the case. The most frequent procedures were bronchoscopy and the placement of a central venous catheter because these patients will require long-term administration of medications.

RSV is the main respiratory pathogen in children under 2 years of age, and reinfection is common. Recent studies have been shown that hospitalizations of patients for respiratory infections in children under 4 years of age were predominantly for disease of viral etiology; of these, only 10% were due to rhinovirus, and the remaining 90% were due to RSV or in combination with rhinovirus and bocavirus [24]. Current guidelines recommend the prevention of RSV diseases with emphasis on hygienic measures and immunoprophylaxis as crucial aspects for reducing the transmission of this pathogen, especially in children with comorbidities [25, 26]. It is worth mentioning that none of the patients included in this study received immunoprophylaxis for RSV, as it is not a drug available for patients attending the IMSS. Overall, compared with other publications [12], this study identifies a longer hospital stay, in-hospital complications and mortality in the group of patients with a history of prematurity, BPD and CHD.

It is important to mention that the sources of data collection include the population that attends the 3 levels of medical care, offering a broad perspective of disease behavior in Mexico.

Our study has limitations. First, patients with CHD and BPD were not classified according to the severity of these diseases, as patients with complex heart disease and severe BPD have a higher risk of complications compared to patients with CHD with low hemodynamic impact or mild BPD. Second, this study was performed with a secondary analysis of a database, whereby the diagnosis of VRI was considered through the codes registered in this database; in Mexico, a diagnosis is usually made based on clinical data and considering that 90% of respiratory airway infections in patients under 2 years of age have a viral etiology. Third, socioeconomic levels were not included in the analysis, though this factor has been shown to impact complications in these patients [27].

Mexico does not have established guidelines for the prevention and etiological diagnosis of lower respiratory infections, which generates a significant delay and an increase in the costs of care for these patients. Therefore, based on this study’s results, we recommend making clinical guidelines identifying the patients with greater complication risks.

## Conclusions

Patients between 1 and 24 months of age hospitalized for VRI with some existing pathology, such as prematurity, BPD and CHD, invasive medical procedures and diagnosis of pneumonia, are most affected by in-hospital complications. Factors associated with the mortality of patients between 1 and 24 months of age hospitalized for VRI were BDP, CHD, invasive medical procedures, in-hospital complications and diagnosis of pneumonia.

Finally, a strategy that should be implemented is the use of inmunoprophylaxis for RSV in patients with prematurity, BPD, and/or CHD to prevent complications in this risk population.

## Data Availability

The datasets generated and/or analysed during the current study are not publicly available due, because it is a secondary analysis of a database obtained to record data from the hospitals affiliated with the IMSS and belongs to the IMSS, but are available from the corresponding author on reasonable request.

## Abbreviations

VRI: Viral respiratory infections
RSV: Respiratory syncytial virus
PICU: Pediatric intensive care unit
BPD: Bronchopulmonary dysplasia
CHD: Congenital heart disease
IMSS: Mexican Social Security Institute
ICD 10: International Classification of Diseases
wGA: weeks of gestational age
CI95%: Confidence interval 95%
